# Six-Month Color Stability Assessment of Two Calcium Silicate-Based Cements Used in Regenerative Endodontic Procedures

**DOI:** 10.3390/jfb10010014

**Published:** 2019-02-28

**Authors:** Paulo J. Palma, Joana A. Marques, Rui I. Falacho, Eder Correia, Alexandra Vinagre, João Miguel Santos, João C. Ramos

**Affiliations:** 1 Institute of Endodontics, Faculty of Medicine, University of Coimbra, 3000-075 Coimbra, Portugal; ppalma@uc.pt (P.J.P.); joanaamarques@hotmail.com (J.A.M.); 2 Institute of Oral Implantology and Prosthodontics, Faculty of Medicine, University of Coimbra, 3000-075 Coimbra, Portugal; rifalacho@fmed.uc.pt; 3 Dentistry Department, Faculty of Medicine, University of Coimbra, 3000-075 Coimbra, Portugal; edercorreiajf@gmail.com; 4 Institute of Operative Dentistry, Faculty of Medicine, University of Coimbra, 3000-075 Coimbra, Portugal; avinagre@yahoo.com (A.V.); jcramos@fmed.uc.pt (J.C.R.)

**Keywords:** Biodentine, calcium silicate-based cements, mineral trioxide aggregate, regenerative endodontic procedures, tooth discoloration

## Abstract

Aim: The purpose of the present study is to assess the color stability of two calcium silicate-based cements (CSCs) used in regenerative endodontic procedures (REPs). Methods: A total of 40 acrylic single-rooted transparent teeth, with immature apex, were used. Root canals were filled up to 3 mm below the level of the cementoenamel junction, with either saline solution (Mineral Trioxide Aggregate (MTA)/saline and Biodentine/saline) or blood (MTA/blood and Biodentine/blood). Subsequently, ProRoot MTA^®^ or Biodentine^TM^ was placed in the root canal to create a cervical barrier. Color measurement was carried out at four different evaluation periods (3 h, 72 h, 7 days, and 6 months). Shade analysis within the L* a* b* color space was performed and color variation (∆E) calculated. The significance level for statistical analysis was set at *p* < 0.05. Results: The four groups showed a significant decrease in L* values over time. The ΔE value increased over time for all groups but was not statistically significant for the Biodentine/blood group. Two-way ANOVA showed no interaction between the CSC and treatment (contact with saline solution or blood). CSC used was the factor responsible for ΔE over time, inducing statistically significant color variations from T_3H_ to T_7D_ (*p* = 0.04) and T_3H_ to T_6M_ (*p* < 0.01). After 6 months, MTA/saline had 5.08 (*p* = 0.001) higher ΔE than Biodentine/Saline and the MTA/blood had 3.65 (*p* = 0.009) higher than Biodentine/blood. Conclusions: After 6 months, regardless of blood exposure, Biodentine exhibits superior color stability compared to MTA. Biodentine might be a suitable alternative to MTA as a cervical barrier material in REPs.

## 1. Introduction

Tooth discoloration is a major esthetic concern for both patient and dental practitioners, especially when anterior teeth are involved [1,2]. Tooth staining exhibits a multifactorial etiology, with localized intrinsic discoloration appearing to be, in some clinical situations, related to both intra and post-endodontic procedures [2].

Concerning the clinical management of immature permanent teeth with pulp necrosis apexification induces an apical barrier formation, formerly with a multiple-visit calcium hydroxide application [3] but more recently with a mineral trioxide aggregate (MTA) apical plug placement [4]. High success rates were reported regarding the aforementioned apical MTA barrier technique [5]. However, considering the well-known drawbacks inherent to the conventional approach, regenerative endodontic procedures (REPs) have been presented as a viable biologically based treatment alternative [6,7], aiming to promote the continuation of root development in both length and width of the root walls, with the ultimate goal of reestablishing a positive pulp vitality test response [8,9,10,11]. According to the current American Association of Endodontists (AAE) Clinical Considerations for a Regenerative Procedure, following a meticulous disinfection protocol, a scaffold is created/positioned within the root canal space [10]. Despite its limitations and the existence of several alternatives, such as platelet-rich plasma, platelet-rich fibrin, and soluble collagen, a blood clot creation is advised by the AAE [10] and has become traditionally used as a scaffold, with clinical studies highlighting the success of this approach [12]. Subsequently, cervical sealing is performed by placing a 3–4 mm CSC layer in the coronal portion of the root canal, over the scaffold [10,11].

Tooth discoloration following REPs is well described in the literature [13]. The AAE recommends clinicians to provide informed consent that aims to advise the patient of the possible postoperative adverse effects, including tooth staining [10]. This undesirable outcome arises from the use of specific endodontic materials with discoloration potential in regenerative therapy [14]. Since MTA discoloration potential has been stated as a main shortcoming, an alternative bioactive cement, exhibiting similar clinical applications and a shorter setting time (12 min), was introduced—Biodentine (Septodont, Saint-Maur-des-Fossés Cedex, France) [15]. This bioceramic material contains zirconium oxide, rather than bismuth oxide that is found in MTA, to provide radiopacity, and previous studies report superior color stability compared to ProRoot MTA^®^ (Dentsply Tulsa Dental, Johnson City, TN, USA) [16,17]. Moreover, it is noteworthy that the presence of blood during calcium silicate-based cement (CSC) setting can exacerbate both MTA and Biodentine color variation over time [18,19,20] and, therefore, impair the esthetic outcome following REPs.

The current study detaches itself from a wide span of others as it considers the multifactorial tooth discoloration process and isolates a single variable, blood exposure influence, to clearly assess its role. To increase reproducibility and external validity, an acrylic immature tooth model was used to simulate the clinical situation of a permanent tooth eligible for being used in regenerative endodontic procedures.

The purpose of the present in vitro study was to assess the color stability of two CSCs (ProRoot MTA^®^ and Biodentine) used in regenerative therapy, in the presence and absence of blood and at four different timings, using an experimental model for REPs simulation.

The experimental setup tested the null hypothesis that no statistically significant differences would be found in the variation of the mean *Commission International de l’Eclairage* (CIE) L* a* b* color coordinates of the cervical barrier, made with two different calcium silicate-based cements, either in contact with saline solution or blood.

## 2. Results

**Figure 1:**
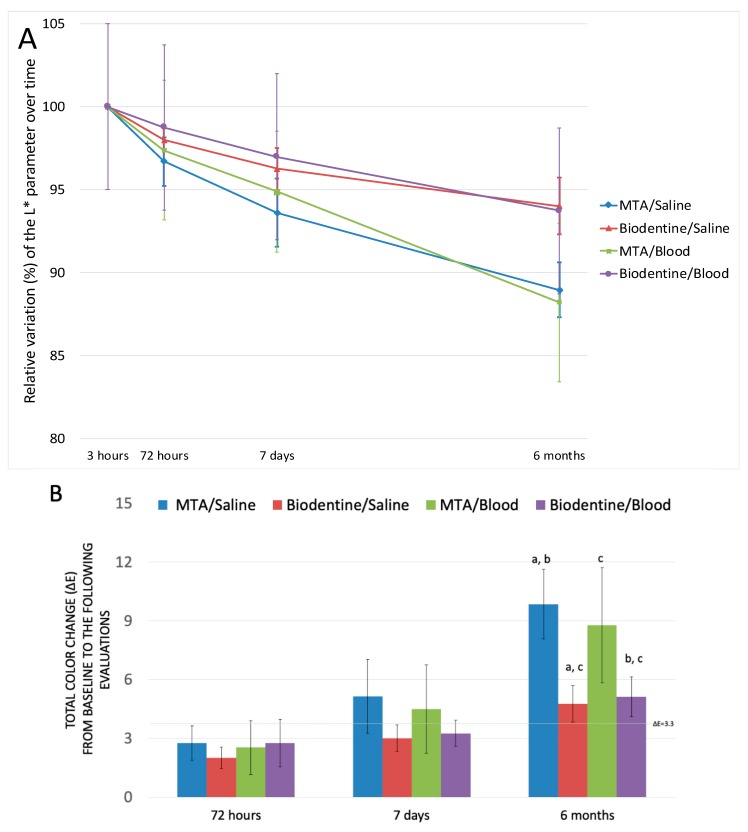
Color variation of the cervical barriers for all groups and assessment periods. (**A**) Relative variation of *Commission International de l’Eclairage* (CIE) parameter L* value of groups measured at T_3H_, T_72H_, T_7D_, and T_6M_. The relative variation of lightness decreases significantly in all groups over time. The vertical lines represent the 95% CI. (**B**) Graphical representation of the total color variation (ΔE) from T_3H_ to each of the following evaluations. The vertical lines represent the 95% CI. One-way ANOVA did not detect significant differences between groups for ΔE T_3H_ − T_72H_ (*p* = 0.724) and for ΔE T_3H_ − T_7D_ (*p* = 0.198). After 6 months, statistically significant differences were found between groups for ΔE T_3H_ − T_72H_ (*p* = 0.001). Similar superscript letters denote pairs of groups that differ at the 0.05 level. MTA, mineral trioxide aggregate.

Table 1 presents the descriptive statistics of the mean CIE L* a* b* coordinates of the cervical barrier for all groups at each period of evaluation (T_3H_, T_72H_, T_7D_, and T_6M_). Due to the inherent color of the CSC materials, three hours post-compaction of the barriers against saline solution, MTA presented L* values statistically higher than Biodentine (*p* = 0.005), whereas no statistically significant differences could be detected between the blood-compacted groups (*p* = 0.312). The four groups showed a significant decrease in L* values over time. While Biodentine samples revealed a constant decrease in lightness, the samples with MTA revealed an intense reduction in the L* values from T_7D_ to T_6M_, regardless of the treatment (saline or blood). For this reason, MTA showed the highest decrease from T_3H_ to T_6M_ with mean differences of 8.67 (95% CI: [5.87–11.45]) and 8.57 (95% CI: [3.73–13.41]), respectively, for the samples in contact with saline and blood. The lightness of Biodentine samples decreased 4.41 (95% CI: [2.79–6.03]) and 4.46 (95% CI: [2.59–6.33]) points, respectively, when in contact with saline or blood. The kinetic analysis of the relative variation of the L* parameter over time (3 h to 6 m) shows superior variation for MTA groups when compared to the Biodentine groups (Figure 1A).

MTA barriers presented neutral values within the blue/magenta channel (a*) in all periods of evaluation, regardless of the treatment, whereas Biodentine barriers present a slight deviation towards the positive (red) portion of the spectrum. In all groups, the b* values were located within the positive portion of the spectrum, conferring a yellowish aspect to the barriers, which was more noticeable in the Biodentine samples. Though significant in some cases (Table 1), the variations of the color channels a* and b* were not significant within the complete spectrum (Supplementary File 1).

The total ΔE from T_3H_ to each of the following evaluations is presented in Figure 1b. Color variation increased over time for all groups but was not statistically significant for Biodentine/blood group. Two-way analysis of variance determined no interaction between the barrier material and the treatment (Table 2).

Treatment (saline or blood) had no influence on ΔE in any of the intervals evaluated (Table 2). The CSC used was the factor responsible for ΔE over time, inducing statistically significant color variations from T_3H_ to T_7D_ (*p* = 0.04) and T_3H_ to T_6M_ (*p* < 0.01). After 6 months, MTA samples in contact with saline had 5.08 (95% CI: [2.38–7.84], *p* = 0.001) higher ΔE than the corresponding samples of Biodentine, and the MTA/blood had 3.65 (95% CI: [0.95–6.35], *p* = 0.009) higher ΔE than Biodentine/blood. MTA had the highest color variation.

At 6 months all groups presented perceptible color changes (ΔE > 3.3), statistically significant at the α = 0.01 level.

## 3. Discussion

Tooth discoloration following REPs has been described as an undesirable consequence and a valuable patient-centered outcome that affects quality of life when anterior teeth are involved [13]. Treatment, planning, and selection must rely on adequate knowledge of the potential discoloration etiological factors, as well as the underlying mechanisms, in order to achieve endodontic therapy success regarding biological, functional, and esthetical aspects [18]. The purpose of the present study was to assess the color stability of both MTA and Biodentine, over a 6-month period and, therefore, unveil the role played by blood exposure on discoloration severity. This matter holds high clinical significance since blood exposure is likely to occur in several endodontic procedures, including cervical sealing performed in REPs [20,21].

Previous studies reported blood contamination as a factor that exacerbates discoloration in calcium silicate-based materials [18,19,20,21], with bismuth oxide-free Portland cement also presenting color alteration subsequent to blood exposure [18]. The hypothesis of unset MTA presenting surface porosities, which uptake blood elements, has been formulated to explain CSC discoloration enhancement, as the long hydration process inherent to MTA may allow erythrocyte sorption and their further hemolysis, ultimately leading to material and tooth discoloration [18,19]. Contrariwise, Biodentine exhibits a shorter setting time of approximately 12 min [22]. The faster setting of this hydraulic material might present an explanation for the superior color stability of the Biodentine/blood group, when compared to MTA/blood group, since it may limit blood absorption. This hypothesis was proposed in previous studies which also found greater color stability with Biodentine [23]. Recently, a new CSC material (PulpGuard, Coltène/Whaledent, Altstätten, Switzerland) was introduced, showing a record setting time of 3 min, with previous studies confirming a favorable cytocompatibility profile [24]. Another suggested mechanism for tooth discoloration following MTA placement may be expounded by the oxidation and incorporation of the remaining iron content in the set material [19]. Hence, over time, contact with blood triggers loss of the ferrous (Fe^2+^) ion contained within the center of the porphyrin ring through a natural redox reaction that originates Fe^3+^, a dark brown component prone to promote material and, consequently, tooth discoloration [19]. Guimarães et al. researched the effect of propylene glycol in MTA color variation and found the 80% distilled water/20% propylene glycol vehicle significantly lowered the degree of color change when blood exposure occurred [20]. Furthermore, the penetration of blood into tooth structure, with hemoglobin or hematin molecules present within the dentinal tubules, may induce discoloration [25].

Since discoloration results obtained from ex vivo or in vivo models may arise from both material and tooth discoloration influence when in contact with blood, the present study was conducted using an in vitro acrylic model, which allows for solely analyzing the role played by blood–CSC interaction in the color stability of the biomaterials. Furthermore, the experimental model used in the present study aims to anatomically simulate a permanent immature tooth eligible for performing regenerative endodontic procedures on. Regarding sample preparation and, likewise, revascularization protocols [26], root canals were filled up with blood (grouped by MTA/blood and Biodentine/blood) and then a cervical barrier was created. Although the applied protocol mimics the clinical routine, we speculate that MTA and Biodentine discoloration severity may be enhanced by the placement and condensation of CSC against the blood already delivered into the root canal. It is crucial to ensure complete hemostasis [21] and perform adequate blood clot stabilization, as well as to clean the blood present on dentin walls as meticulously as possible, to prevent blood influx into the tooth structure prior to CSC placement within the coronal portion of the root canal.

Regarding the composition of the biomaterial used as a cervical plug in REPs, MTA comprises bismuth oxide (Bi_2_O_3_) as a radiopacifier [27,28]. Previous studies show that MTA can lead to tooth discoloration [14,18,19,29], with data confirming both the hypothesis of bismuth (a) changing from its oxide form to metal by reduction, resulting in a black compound and subsequent tooth staining, or (b) undergoing oxidation when in contact with a strong oxidizing agent (namely sodium hypochlorite and amino acids from dentin matrix collagen) and producing bismuth carbonate, which results in a black precipitate when exposed to light [30,31,32].

Regarding environmental influence, Vallés et al. [33] suggest that bismuth oxide presents oxygen and light sensitivity. In the current study, sample preparation was performed in simultaneous aerobic conditions with exposure to environment light, with all specimens being subsequently stored in the absence of light. Since the conditions were not verified throughout the experimental period of analysis, the potential influence of oxygen and light on the obtained results was lessened.

It is noteworthy that CSCs presenting alternative agents to provide radiopacity within their formula, such as Biodentine containing zirconium oxide (ZrO_2_) rather than bismuth oxide, consistently exhibit superior color stability in previous studies [14,16,17,34], which agrees with our findings.

In this study, color variation increased over time for all groups but was not statistically significant for Biodentine/blood group. However, the four groups showed a significant decrease in L* values over time, with MTA samples revealing late darkening associated with intense reduction in the L* values from T_7D_ to T_6M_, regardless of the liquid treatment (blood or saline solution). Two-way analysis of variance showed no interaction between the CSC and blood/saline. Furthermore, we found that treatment had no influence on color variation in any of the intervals evaluated. In fact, the CSC used was the factor responsible for ΔE over time, with MTA presenting the highest color variation. Our findings highlight the main role played by the material composition, rather than the blood exposure, in discoloration. However, in this model, the use of a gelatin matrix to resemble a typical regeneration procedure could absorb blood, thus reducing the available amount of blood in contact with the CSC, hence limiting its effect on discoloration. It is noteworthy that we used the complete plug area to obtain the average L* a* b* coordinates of each sample for shade analysis at all timeframes. Additionally, we analyzed each plug by thirds, as follows: apical third, median third, and coronal third. In those three cases, the results did not vary between groups. Naturally, the apical third presented higher color variation in all samples and the remaining two-thirds presented the highest color stability. Nonetheless, it is important to mention, once more, that selecting any of the thirds did not modify the results.

Ultimately, it should also be deemed that other variables not accounted for in this experimental study might play a noteworthy role on tooth discoloration. Nevertheless, the material used remains a key factor to consider in the decision-making process.

Regarding long-term success of endodontic procedures, including REPs, adequate coronal sealing is mandatory [35]. Besides our results reporting a lower discoloration degree regardless of the contact with blood, previous studies show that Biodentine might allow immediate (12 min after biomaterial placement) restoration, while restorative procedures performed directly on top of MTA may preferably be performed in a delayed timeframe [22]. Further clinical studies are needed to confirm these results and to provide information regarding the exact mechanisms inherent to CSC and subsequent tooth discoloration.

## 4. Materials and Methods

### 4.1. Experimental Groups

A total of 40 acrylic single-rooted transparent teeth (model 2111-102-PCL2, DRSK, Hässleholm, Sweden), presenting both occlusal access cavity and immature apex, were used in the present study with the ultimate goal of providing a simulation model of permanent teeth eligible for performing regenerative endodontic procedures on, inspired by a previously described model [26].

The artificial teeth were randomly divided in two main groups according to the CSC used to perform cervical sealing: ProRoot MTA^®^ or Biodentine.

Each one of the main groups was secondarily divided in two groups (*n* = 10) depending on the filling of the root canal with either saline solution (groups of MTA/saline and Biodentine/saline) or blood (groups of MTA/blood and Biodentine/blood).

### 4.2. Sample Preparation

The apical opening of the acrylic teeth was first sealed with sticky wax in order to avoid extrusion. Informed consent was obtained from one participant, according to the approval of the Ethical Committee of IRB of the Faculty of Medicine—University of Coimbra (notification CE001/2013), and blood was collected to (a) determine hematocrit value (46.4%) and (b) to fill the root canal space of specimens of blood groups. Root canals were filled up to 3 mm below the level of the cement–enamel junction (CEJ) with either 7 µL of saline solution (MTA/saline and Biodentine/saline groups) or blood (MTA/blood and Biodentine/blood groups).

Subsequently, a 2 mm^3^ absorbable hemostatic gelatin matrix (Spongostan Dental, MS0005, Ethicon Inc., New Jersey, EUA) was placed in the root canal 3 mm apical to the level of the CEJ.

ProRoot MTA^®^ (MTA/saline and MTA/blood groups) or Biodentine (Biodentine/saline and Biodentine/blood groups) were then prepared according a previous study [22]. CSC application was performed to create a 3 mm cervical barrier under 16× magnification with a dental microscope (Leica M320 F12, Leica Microsystems, Werzlar, Germany).

**Figure 2:**
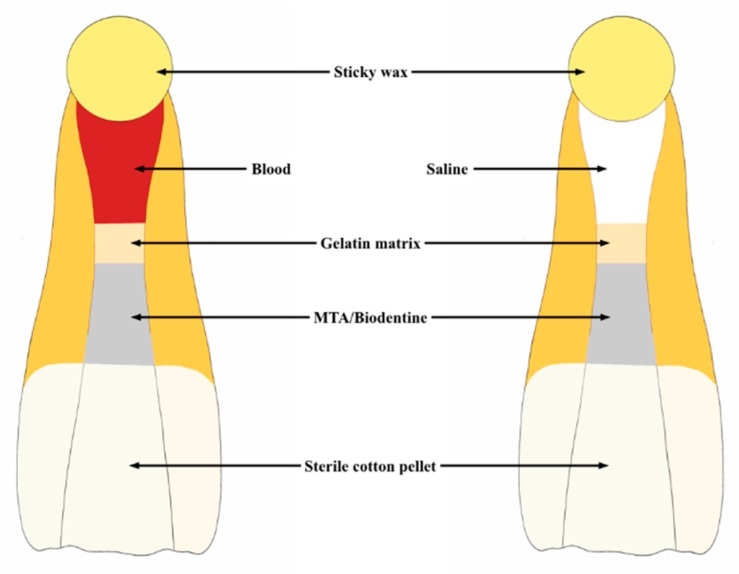
Schematic representation of the experimental setup and storing teeth position.

A sterile saline (sodium chloride 0.9%, Labesfal, Santiago de Besteiros, Portugal) wet cotton pellet was applied over the biomaterial and teeth standardly positioned with crowns facing downwards (Figure 2). All specimens were stored in a dark environment, in an incubator (Gallenkamp, London, UK) at 37 °C and 100% relative humidity throughout the experimental period.

### 4.3. Photographic Record

**Figure 3:**
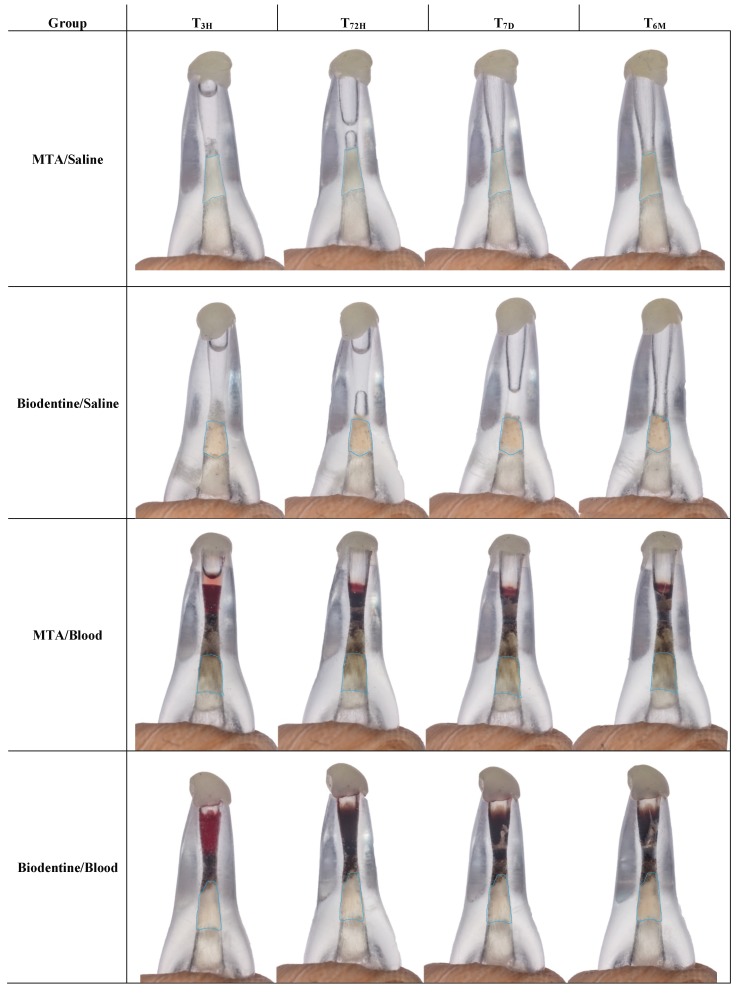
Color assessment of randomly selected representative samples from each experimental group at the four evaluated timeframes (T_3H_, T_72H_, T_7D_, and T_6M_). The blue line represents the total CSC area considered for the shade analysis within the *Commission International de l’Eclairage* (CIE) L* a* b* color space for all timeframes.

The photographic record of the samples was taken with a Canon EOS 5DsR camera using a Canon MP-E 65 mm Macro Lens and a ring flash with cross polarized filters. The settings used were 2× magnification on the manual lens, 1/125 shutter speed, F16 aperture, ISO 100, custom white balance with a 18% grey card, and RAW file format for saving pictures, as seen in Figure 3. Standard positioning of the samples was accomplished by developing a custom-made arm with a silicone (Virtual Refill Putty Fast Set, Ivoclar Vivadent AG, Schaan, Liechtenstein) tip, matching the incisal edge of the acrylic models.

### 4.4. Color Assessment

Color measurement was carried out at four different evaluation periods after sample preparation as follows: (1) T_3H_: 3 h, (2) T_72H_: 72 h, (3) T_7D_: 7 days, and (4) T_6M_: 6 months. Shade analysis within the *Commission International de l’Eclairage* (CIE) L* a* b* color space was performed using ImageJ software (ImageJ v1.52; National Institutes of Health, Bethesda, MD, USA). L* (which corresponds to lightness, ranging from 0 = black to 100 = white), a* (which includes color components from (−) green up to (+) red), and b* (which includes color components from (−) blue up to (+) yellow) values were obtained considering the total CSC area (the complete cervical barrier region was selected and parametric L*, a*, and b* medium values were estimated by the software for each specimen). Subsequently, color variation (∆E) was calculated by using the following formula:

∆E = [(∆L)^2^ + (∆a)^2^ + (∆b)^2^]^1/2^.

### 4.5. Statistical Analysis

Statistical analysis was performed using IBM SPSS v.23 software. Evaluation of the L* a* b* variables of each group in the four periods was performed with repeated ANOVA measures, considering the material and treatment as fixed factors. Assessments of color changes between the first measurement (T_3H_) and each of the following periods of evaluation (T_72H,_ T_7D_, and T_6M_) were made using two-way ANOVA. Group comparisons for each ∆E were performed with one-way ANOVA and Bonferroni post hoc comparisons. Assessments of ∆E within each group were made using the non-parametric Friedman test. ∆E was deemed to be perceptible by the human eye upon comparison with the 3.3 threshold value [34] using the one sample t-test (one-tail). The significance level was set at α = 0.05.

## 5. Conclusions

The choice of the material for cervical barriers must consider not only the clinical and radiographic outcomes, but also the esthetic results. In this case, additional concerns are raised due to the possibility of contact with blood, with unpredictable staining of the cervical barrier and, consequently, of the dental structure. This study demonstrates that the contact with blood does not modify the color alterations suffered by the calcium silicate-based cements after a 6-month period. Within the two materials, Biodentine exhibits superior color stability than MTA.

## Figures and Tables

**Table 1:** Mean values and standard deviation of each of the L* a* b* coordinates of each group for all periods of evaluation.

Groups	Coordinates	T_3H_	T_72H_	T_7D_	T_6M_	*p*
MTA/Saline	L*	78.52 ± 1.58	75.95 ± 1.79	73.51 ± 2.43	69.86 ± 1.88	<0.01 *
	a*	−0.68 ± 0.10	−0.56 ± 0.14	0.05 ± 0.22	0.78 ± 0.78	<0.01 *
	b*	5.15 ± 0.36	5.53 ± 0.78	5.51 ± 0.59	8.37 ± 3.28	0.016 *
Biodentine/Saline	L*	73.87 ± 1.37	72.39 ± 0.84	71.11 ± 1.42	69.46 ± 1.92	<0.01 *
	a*	2.88 ± 0.16	3.12 ± 0.25	3.22 ± 0.27	3.36 ± 0.29	0.003 *
	b*	11.12 ± 0.92	11.89 ± 1.01	11.90 ± 1.13	12.21 ± 0.98	0.0046
MTA/Blood	L*	72.79 ± 5.70	70.86 ± 4.81	69.07 ± 4.06	64.22 ± 4.95	<0.001 *
	a*	0.20 ± 0.63	−0.02 ±0.037	0.41 ± 0.49	0.41 ± 0.64	0.047
	b*	7.14 ± 2.78	7.44 ± 2.73	7.80 ± 2.92	7.65 ± 2.24	0.593
Biodentine/Blood	L*	71.21 ± 3.22	70.33 ± 3.98	69.07 ± 4.10	66.75 ± 3.38	<0.01 *
	a*	2.92 ± 0.23	2.48 ± 0.48	2.69 ± 0.55	2.88 ± 0.37	0.128
	b*	9.32 ± 0.93	8.77 ± 2.56	9.33 ± 1.78	11.05 ±1.65	0.035 *

* Statistically significant different (*p* < 0.05). MTA, mineral trioxide aggregate.

**Table 2:** Two-way analysis of variance of the color variation of the samples, from baseline to each evaluation point (ΔE value ± standard deviation).

	MTA/Saline	Biodentine/Saline	MTA/Blood	Biodentine/Blood	Two-Way ANOVA		
					Factor Material	Factor Treatment	Interaction (Material * Treatment)
**ΔE T_3H_ − T_72H_**	2.76 ± 1.43	2.00 ± 0.88	2.53 ± 2.23	2.76 ± 1.96	0.625	0.629	0.364
**ΔE T_3H_ − T_7D_**	5.14 ± 3.03	3.01 ± 1.10	4.49 ± 3.65	3.26 ± 1.07	0.040 *	0.798	0.568
**ΔE T_3H_ − T_6M_**	9.84 ± 2.67	4.76 ± 1.48	8.77 ± 4.73	5.12 ± 1.63	<0.01 *	0.708	0.452
**Friedman test**	<0.01 *	0.01 *	<0.01 *	0.082			

Each evaluation point (ΔE value ± standard deviation). * Statistically significant different (*p* < 0.05).

## Supplementary Materials

The following are available online at https://www.mdpi.com/2079-4983/10/1/14/s1.
